# Relevance of carbon stocks of marine sediments for national greenhouse gas inventories of maritime nations

**DOI:** 10.1186/s13021-017-0077-x

**Published:** 2017-05-10

**Authors:** Silvania Avelar, Tessa S. van der Voort, Timothy I. Eglinton

**Affiliations:** 1 Geological Institute, ETH Zurich, Sonneggstr. 5, 8092 Zurich, Switzerland; 2 Marine Chemistry & Geochemistry Department, Woods Hole Oceanographic Institution, Woods Hole, MA 02543 USA

**Keywords:** Carbon stocks, Sediments, Oceans, Climate change, Exclusive Economic Zone, Carbon inventory

## Abstract

**Background:**

Determining national carbon stocks is essential in the framework of ongoing climate change mitigation actions. Presently, assessment of carbon stocks in the context of greenhouse gas (GHG)-reporting on a nation-by-nation basis focuses on the terrestrial realm, i.e., carbon held in living plant biomass and soils, and on potential changes in these stocks in response to anthropogenic activities. However, while the ocean and underlying sediments store substantial quantities of carbon, this pool is presently not considered in the context of national inventories. The ongoing disturbances to both terrestrial and marine ecosystems as a consequence of food production, pollution, climate change and other factors, as well as alteration of linkages and C-exchange between continental and oceanic realms, highlight the need for a better understanding of the quantity and vulnerability of carbon stocks in both systems. We present a preliminary comparison of the stocks of organic carbon held in continental margin sediments within the Exclusive Economic Zone of maritime nations with those in their soils. Our study focuses on Namibia, where there is a wealth of marine sediment data, and draws comparisons with sediment data from two other countries with different characteristics, which are Pakistan and the United Kingdom.

**Results:**

Results indicate that marine sediment carbon stocks in maritime nations can be similar in magnitude to those of soils. Therefore, if human activities in these areas are managed, carbon stocks in the oceanic realm—particularly over continental margins—could be considered as part of national GHG inventories.

**Conclusions:**

This study shows that marine sediment organic carbon stocks can be equal in size or exceed terrestrial carbon stocks of maritime nations. This provides motivation both for improved assessment of sedimentary carbon inventories and for reevaluation of the way that carbon stocks are assessed and valued. The latter carries potential implications for the management of human activities on coastal environments and for their GHG inventories.

**Electronic supplementary material:**

The online version of this article (doi:10.1186/s13021-017-0077-x) contains supplementary material, which is available to authorized users.

## Background

### Global carbon cycle dynamics

**Fig. 1:**
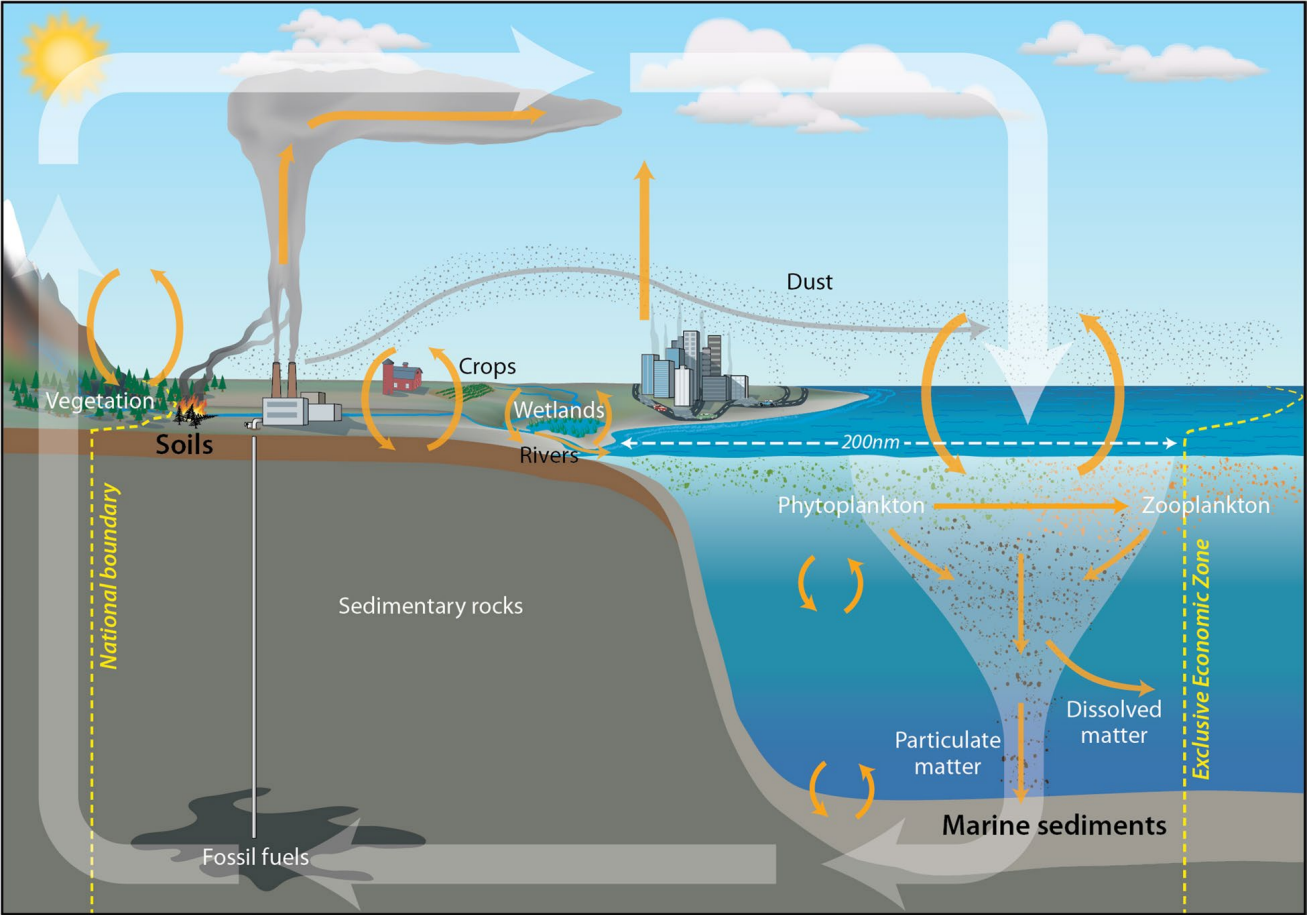
General representation of the global carbon cycle (credit to Jack Cook, Woods Hole Oceanographic Institution). The broad translucent* white arrows* illustrate the general motion of the carbon cycle, depicting production and burial of carbon, and its uplift erosion and weathering resulting of return of this carbon to the atmosphere. The* orange arrows* highlight loci of carbon (CO2) exchange and transfer, such as via photosynthesis and respiration, including examples of human influences on carbon cycle processes. A hypothetical national boundary and exclusive economic zone (EEZ) are indicated as* yellow dashed lines*

Carbon forms the basic building block of life on Earth, and is stored in the atmosphere, land and ocean. In the atmosphere, carbon is contained in greenhouse gases, such as carbon dioxide and methane, where it exerts a controlling role on climate. Terrestrial and aquatic plants remove carbon from the atmosphere through photosynthesis and produce reduced (organic) carbon. Carbon-based food is consumed by animals, which respire carbon dioxide. When plants and animals die and decay, the majority of carbon is remineralized and transformed into inorganic carbon in atmospheric or oceanic reservoirs. A minor portion of this carbon is transferred to underlying soils and sediments, where it has the potential to be sequestered indefinitely. Furthermore, reduced carbon fixed on land may be exported by fluvial and atmospheric processes to the aquatic realm, evolving as it moves through along a complex land–ocean continuum, with some fraction of this carbon ultimately contributing to the oceanic carbon reservoirs. This myriad of processes is called carbon cycle (see Fig. 1). The carbon cycle operates on a broad range of spatial and temporal scales, and involves highly complex and dynamic interactions within as well as at the interfaces between atmospheric, terrestrial and aquatic spheres [17, 23].

Terrestrial ecosystems include living organisms, litter and soils, with the latter representing the largest terrestrial carbon reservoir [4]. Terrestrial material is transported to oceans via eolian and fluvial processes. Inland waters such as rivers, lakes and wetlands are seen as key components in this “boundless carbon cycle”, that mediate the lateral and vertical carbon fluxes between land, ocean and atmosphere [6]. Drainage basin properties such as catchment size, morphology, geology, vegetation, soil and rock erosion, weathering, as well as human influences such as dams and land-use, influence the flux and nature of organic carbon exported by rivers [45]. Even though inland waters cover a small part of the terrestrial surface, they sequester equal amounts of carbon as their watersheds, and recycle and respire large amounts of terrestrial carbon [3].

Coastal environments including wetlands, estuaries, reefs and bays host diverse ecosystems, e.g., mangroves, salt marshes, sea grasses, serve as a link between the terrestrial and marine realms, and are recognized as important, yet vulnerable, carbon sinks (so-called “blue carbon” [40]). Coastal ecosystems cover just 2% of the Earth’s surface, but are responsible for around 50% of the transfer of carbon to sediments and sequester carbon more efficiently than terrestrial ecosystems of equivalent area [31]. Besides the large carbon storage potential in soils and sediments of these coastal areas, they provide a unique habitat for aquatic life and harbor enormous biodiversity, whilst also contributing to shoreline protection and pollution attenuation.

In the pelagic marine realm, ecosystems are fueled by primary production by phytoplankton in the surface ocean that fixes inorganic carbon, and converts it to particulate and dissolved organic carbon. These closely linked carbon pools undergo complex transformations, many of which are biologically mediated, with a portion of this recently fixed carbon escaping from surface waters and being exported to deep water and underlying sediments via the so-called “biological pump” [23, 35].

As a result of long-term operation of this complex network of carbon cycle processes, ocean floor sediments and associated sedimentary rocks constitute the largest reservoir of carbon on the planet. The vast majority (approximately 90%) of sequestered organic carbon accumulates on the continental margins, with deltaic environments and fjords as prominent loci of carbon burial (e.g. [22, 52]).

About one-third of all human-generated carbon emissions are dissolved in the ocean. Direct anthropogenic intervention and human-induced climatic change have altered the carbon and nutrient cycling throughout the land–ocean aquatic continuum [30, 46]. By cycling and retaining huge amounts of carbon, oceans help to regulate the Earth’s climate and alleviate the impacts of global warming on the environment (see Fig. 1). However, given carbon emissions at unprecedented rates [27], and attainment of concentrations that have not been witnessed in at least the past several hundred thousand years [43], understanding the partitioning of this carbon between atmospheric, terrestrial and oceanic reservoirs is brought into sharp focus. The disturbance of the carbon cycle and consequent destabilization of the global climate has now become a societal and economic issue [36, 41]. In addition to climate change on a global scale that is now recognized to be underway [27], the asymmetric geographic distribution of factors contributing to this change, those who are experiencing its consequences, and those with financial resources to influence future change or implement compensatory measures, leads to complex international tensions.

### Carbon stocks and inventories

Carbon stocks convey the amount of carbon contained in a reservoir or system in relation to its capacity to accumulate or release carbon. The calculation of carbon stocks has been based on carbon reservoirs associated with terrestrial ecosystems, anthropogenic sources and the atmosphere. Carbon inventory processes are inherently linked with anthropogenic impacts on the environment. The methodological approach entails a multiplication of human activity (or activity data) with emissions or removals per unit activity (emission factors) [47]. Thus, coupled to the assessment of the size of the carbon stocks, there should be an assessment and assertion of the anthropogenic impact on corresponding reservoir (e.g., soils) within the national boundaries.

Terrestrial carbon accounting, particularly regarding forest carbon and associated risks, is well developed (e.g. [24, 62]). In contrast, frameworks for assessment of anthropogenic impact on marine carbon stocks are not developed, and are beyond the scope of this paper. The current carbon accounting framework does not consider human activities in the marine realm [53], despite the integral role of the oceans in the global carbon cycle and their vulnerability to climate change. Several human activities that impact carbon stocks on land and are included in terrestrial inventories [48] also take place in the shallow coastal oceans and may directly impact and potentially endanger coastal carbon stocks [42]. These include fishing and fish farming, as well as mining and excavation (dredging). There are also other more indirect consequences of human activity that may perturb marine carbon inventories [30], such as changes in fluvial sediment delivery to ocean [55], as well as climate change-driven ocean warming, deoxygenation, and acidification [20]. Overall, we posit that, given (i) the magnitude of ocean carbon stocks, (ii) direct and indirect human influences on coastal environments and underlying sediments, and (iii) the precedent set by including wetlands in NGGI [28], there is a need to better constrain oceanic carbon stocks and to evaluate whether they should be included in national carbon inventories.

Significant information gaps exist with respect to carbon stocks in terrestrial and marine ecosystems, both in the context of C inventories in biomass, soil and sediments, as well as regarding potential disturbance, accumulation or attrition of these stocks. It is challenging to develop global-scale assessments of organic matter in oceans, as they include inactive pools stored in sediments and fossil deposits and bioactive reservoirs such as dissolved CO_2_ and H_2_CO_3_, marine organisms that differ markedly in rates of turnover and hence influence on atmospheric C inventories [23]. At present, estimates of the amount and global distribution of organic carbon residing within ocean sediments present an extremely simplified, coarse picture of spatial variability in content, and especially carbon type. In particular, neritic and coastal environments—the nexus between land and deep ocean—are both particularly important in the context of both marine resources and carbon stocks, and highly vulnerable to change [7]. Yet these environments are understudied from the perspective of carbon stocks relative to their terrestrial counterparts.

Assessment of terrestrial carbon stocks has been an important research and legislative focus over several years. For example, the rapid carbon stock appraisal (RACSAL) method is used to estimate changes in carbon stock within and between land-cover classes of land use systems, which refer to vegetation types, human activities and land-cover changes [21], so it focuses exclusively on terrestrial ecosystems. Ajani [1] raised the question of how carbon stocks in the oceans can be included in the environmental economical accounting. Regnier et al. [46] suggested that carbon fluxes along the land–ocean aquatic continuum should be included in global carbon dioxide budgets. Ajani et al. [2] proposed a comprehensive carbon accounting framework that includes stocks as well as flows for reservoirs, lands and activities continuously over time. The framework differentiates carbon reservoirs in the geosphere, biosphere and anthropogenic, but not the oceans. We suggest that assessment of carbon stocks of continental margin sediments within the Exclusive Economic Zones (EEZ) of maritime nations may provide a useful framework for greenhouse gas emissions accounting, and for developing strategies for coastal carbon management as part of planning long-term responses to climate change. A preliminary study [38] estimated that carbon uptake within the EEZ of Australia accounted for 30–40% of total Australian emissions in the 1990s.

In order to examine sediment C stocks in the context of those in soils on the adjacent land surface, and in relation to national boundaries, we define areal extents based on marine EEZs as we believe this may serve as a useful framework for assessment and discussion of inventories in the context of climate change mitigation polices. Current international agreements on EEZs and climate change are summarized in the supplemental material. In the following section, we introduce a method for the estimation of organic carbon stock per unit area where the ocean is considered. We then describe the data used to estimate carbon stocks of sediments and soils for continental shelves and land areas for our primary test case Namibia, which was chosen based on the wealth of marine sediment carbon data that presently exists. We then extend this approach to two other test countries, Pakistan and the United Kingdom, which have contrasting characteristics in their marine and terrestrial domains. Finally, we compare estimated carbon stocks, present discussions and conclusions.

## Methods

### Estimation of carbon stocks of soils and marine sediments

Stocks and changes are statistical estimates, i.e., they are not accurate values and are frequently associated with uncertainties in measured variables [56]. An approximation of carbon stocks of a country is sufficient to give a general indication of their magnitude in this study. Our approach for estimating carbon stocks requires organic carbon (OC) concentrations (gC/kg) and bulk density (BD) measurements (g/cm^3^) of samples located within a given depth interval in soil or sediment. BD is used to convert organic carbon concentrations to mass per soil area at a chosen depth (Eq. 1). BD values vary with age, depth and state of compaction, where compaction increases BD, and different matrix types have different grain densities and packing geometries. In the present study, carbon stock is calculated as tonnes of carbon per hectare (t/ha), i.e., equivalent to kgC/m^2^, for the top 0-10 cm depth interval of soils/sediments. OC data is described as a percentage (%) of the topsoil, e.g., 3.5 g carbon per 100 g soil or sediment, or 35 g per kg.

1 $${\text{C stock }}\left( {{\text{t}}/{\text{ha}}} \right) \, = \left[ {\text{TOC}} \right]\left( \% \right)\; \times \;{\text{BD }}\left( {{\text{g}}/{\text{cm}}^{ 3} } \right) \times \;{\text{depth }}\left( {\text{cm}} \right)$$

In the present investigation, we do not seek to scrutinize prior assessments of soil carbon stocks, and instead report values based on established sources (e.g., FAO). Our primary focus is to undertake preliminary assessments of organic carbon stocks in sediments that fall within the EEZ of those nations chosen for this exploratory study.

### Case studies: EEZ and organic carbon of Namibia, Pakistan and United Kingdom

**Fig. 2:**
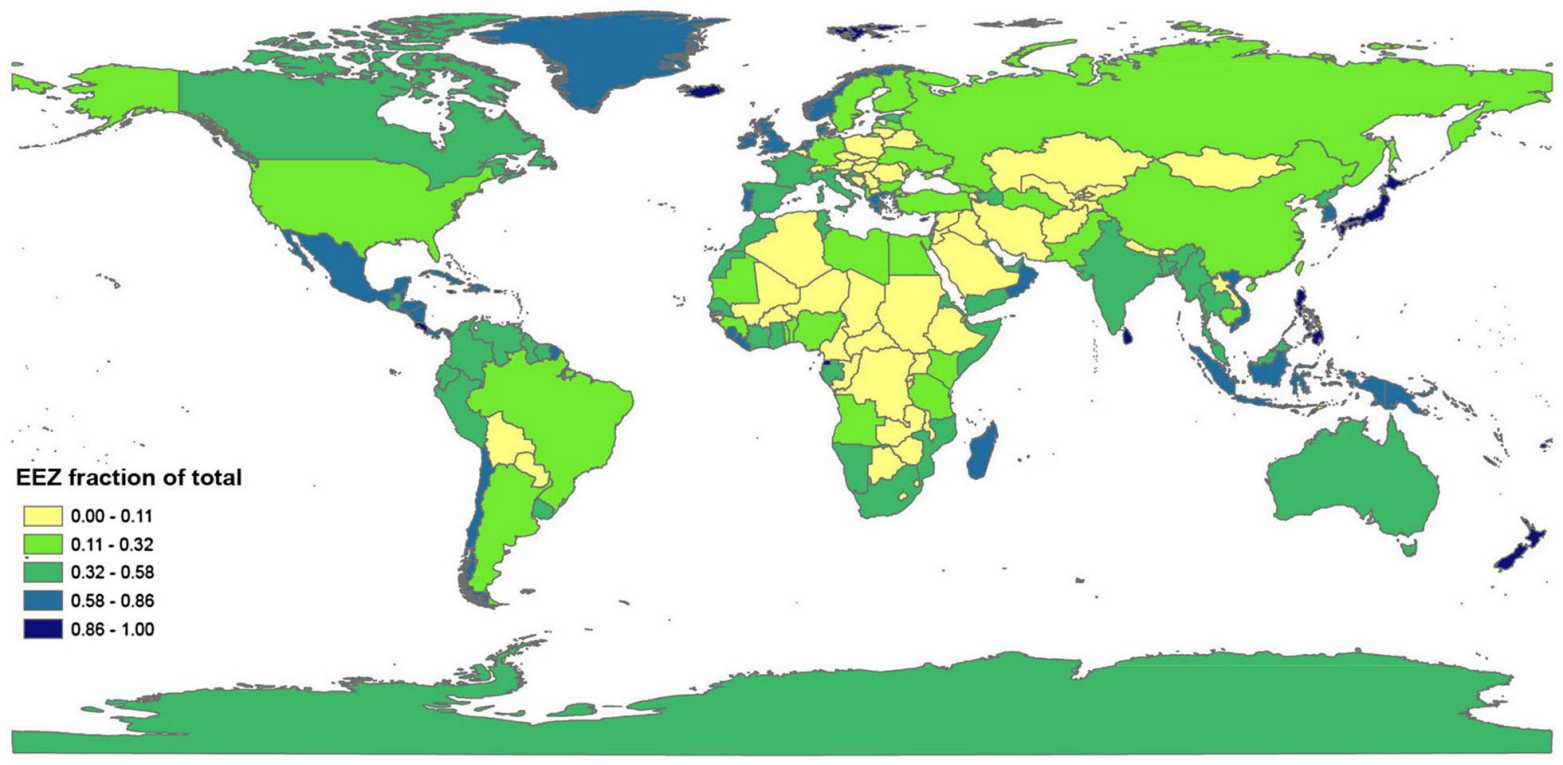
EEZ fraction of total (land plus EEZ) area of nations, considering data and situation in 2016 (see Additional file 1: Table S1)

Our study was carried out with data samples drawn from Namibia, where a strong coastal upwelling system prevails and a wealth of sediment data is available, in order to estimate carbon stocks within its adjacent continental margin sediments and mineral soils. For comparison, we also estimated approximate carbon stocks for sediments and soils of Pakistan and the United Kingdom. These three countries ratified the Kyoto Protocol and have potential activities that could impact sediment carbon storage in their EEZs, if they were managed. These countries were selected also due to their contrasting EEZ-land ratios (see Fig. 2 and Additional file 1: Table S1 that presents EEZ fraction of total area for all maritime nations), location in different continents, marked variations in terrestrial and marine ecosystems and corresponding carbon depositional (soil, sedimentary) environments. For simplicity we did not consider blue carbon (coastal wetlands) estimates here, but blue carbon stores in the coastal environment of the United Kingdom are potentially highly significant (e.g. [10]). Namibia and Pakistan are high OC burial sites due to high productivity from coastal upwelling and pronounced oxygen minimum zones. Their sediments are carbon-rich whereas their soils (mostly deserts) are relatively barren in carbon. Both countries do not have extensive coastal wetland systems, and serve as simplified “binary” systems where soil and marine sediment carbon stocks can be more readily compared.

Prospective data sources include the scientific literature, the geo-reference data library PANGAEA (http://www.pangaea.de), databases of international institutions, e.g., Harmonized World Soil Database (HWSD) [39], ISRIC-WISE [5], LUCAS [57] and Africa DB [32], FAO [15] and governmental institutions. The available data have different coverage, size and release dates. We rely primarily on official data that encompass the required parameters in a consistent way for all three countries.

Measures of OC and BD were chosen for the upper 10 cm layer of soil and sediment, using two significant figures, whenever possible. When a subset of available data spanned a different depth range, the values were proportionally adjusted to the top 10 cm. Mean values of OC concentrations and BD were used as inputs in the estimation of carbon stocks. We utilized established sources and adopted established values for soil OC, in order to focus in estimates for sedimentary inventories of OC within EEZs of Namibia, Pakistan and the United Kingdom [61]. Detailed information about the EEZ, OC and BD of sediments and soils from the three maritime nations are given in the Additional file 1.

## Results

### Data Synthesis

**Fig. 3:**
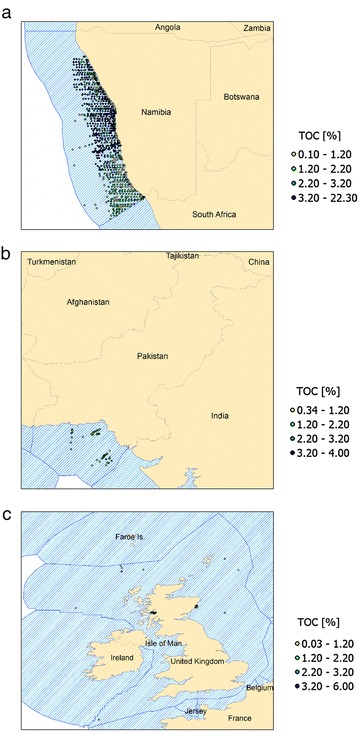
Sediment samples within the EEZ of** a** Namibia [26],** b** Pakistan [11, 12, 49, 54] and** c** United Kingdom [13, 18, 19, 34, 51]

The organic carbon and bulk density values of top soils and sediments from Namibia, Pakistan and the United Kingdom used in the estimation of carbon stocks are summarized, with data sources and statistics, in Table 1. For visualizing the sediment data, we mapped the considered samples within the EEZ of Namibia, Pakistan and United Kingdom, which are shown in Fig. 3.

**Table 1:** Overview of OC and BD data statistics for sediments and soils from Namibia, Pakistan and the United Kingdom

	Namibia	Pakistan	United Kingdom
	Sediment organic carbon		
Sample depth (cm)	0–30 [26]	0–10 [49, 54], 0–2 [11, 12]	0–3 [18, 51], 0–1 [13, 19, 34]
OC_average_ (%)	3.72	1.72	0.78
Number of samples	968	109	102
Min. value	0.1	0.34	0.03
Max. value	22.3	4	6
	Soil Organic Carbon		
Sample depth (cm)	0–10 [15]	0–10 [15]	0–10 [15]
OC_average_ (%)	0.34	0.86	6.98
	Sediment Bulk Density		
Sample depth (cm)	0–10 (inferred) [16]	0–2 [11, 14]	0–15 [8]
Bulk density_average_ (kg/dm^3^)	1.17	0.82	1.13
Number of samples	16	24	137
Max. value	n.a.	1.06	1.5
	Soil Bulk Density		
Sample depth (cm)	0–30 [32]	0–50 [25]	0–15 [60]
Bulk density_average_ (kg/dm^3^)	1.56	1.5	0.73
Number of samples	17	Raster data	1385
Min. value	1.25	1.3	0.15
Max. value	1.76	2.6	1.30

### Estimated carbon stocks

Marine continental margin ecosystems and associated depositional settings [33] are heterogeneous and, although there is typically a general relationship between organic carbon and water depth (deeper waters, lower carbon contents), carbon contents of underlying marine sediments vary greatly. However, measurements of organic carbon in sediments, especially together with bulk density along continental margins, remain surprisingly scarce in the scientific literature (e.g., [50]) and the number of measurements of carbon and other biogeochemical data are generally too limited to provide sufficient coverage for robust estimates over large areas. Therefore, the carbon stocks estimated in this study carry large uncertainties, including from the approach of adopting a statistical mean to represent the collected data, which may artificially bias calculated values. For example, in locations characterized by little available data (e.g., distal regions of the Namibian EEZ) the mean value could be strongly biased or thus unrepresentative of EEZ-wide values. In addition, the estimated carbon stocks of Pakistan and the United Kingdom are not as robust as the stocks of Namibia, because there are less sediment data available for Pakistan and the United Kingdom than for Namibia. Thus, rather than providing the most robust estimates for sediment and soil carbon stocks, the primary goal of this preliminary assessment is to raise the fundamental question concerning the potential integration of marine sediments into carbon stocks and inventories. We consider these crude approximations as sufficient for our purpose, while at the same time as justification for future efforts to acquire more sediment data.

Carbon stocks in the sediments within the corresponding EEZs of Namibia, Pakistan and the United Kingdom and in their soils were estimated for the depth interval 0–10 cm depth. Approximated values of carbon stocks in the three countries are summarized in Table 2.

**Table 2:** Sediment and soil data of Namibia, Pakistan and the United Kingdom with their estimated carbon stocks

	Namibia		Pakistan		United Kingdom	
	EEZ	Land	EEZ	Land	EEZ	Land
	Sediment	Soil	Sediment	Soil	Sediment	Soil
Organic carbon (%)	1.24	0.34	1.72	0.86	0.78	6.98
Bulk density (g/cm^3^)	1.17	1.56	0.82	1.50	1.13	0.73
Depth (cm)	10	10	10	10	10	10
Carbon stock (t/ha)	14.48	5.30	14.14	12.90	8.73	51.05
Area (ha)	56,010,100	82,329,000	27,225,500	77,088,000	75,663,900	24,193,000
Total carbon stock (tons)	811 × 10^6^	437 × 10^6^	385 × 10^6^	994 × 10^6^	661 × 10^6^	124 × 10^7^
Sediment carbon stock relative to soil carbon stock	186%		39%		53%	

**Fig. 4:**
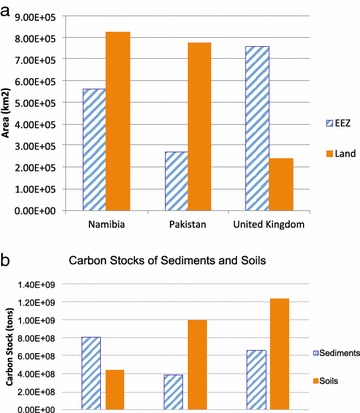
**a** EEZ and land surface area comparison; **b** approximated carbon stocks of sediment and soil organic carbon for Namibia, Pakistan and United Kingdom

Comparing estimates of carbon stocks in continental margin sediments to carbon stocks in soils reveals that organic carbon in Namibian sediments corresponds to 186% of carbon held in its soil. The total maritime zone of Pakistan represents more than 20% of its land area, and sedimentary carbon stocks correspond to almost 40% of the amount of organic carbon in soils of Pakistan. In the United Kingdom, carbon in sediments corresponds to 53% of its soil carbon stock (see Table 2). The proportion of land and EEZ areas for the three countries is shown in Fig. 4a, while the resulting approximated carbon stocks of soils and sediments are shown in Fig. 4b.

## Discussion

Although the EEZ estimates carry substantial uncertainty, being based on relatively sparse OC and/or BD data for marine sediments, they indicate that large stocks of carbon reside in sediments within the EEZs of the three countries examined here. More robust estimates of marine sediment carbon stocks could be derived from (1) additional measurements; (2) use of kriging techniques for more accurate interpolation of data to account for spatial gradients (e.g. [29]); and (3) incorporation of diagenetic models and global observations (e.g. [37]) to better define general trends in OC attenuation with sediment depth.

Nevertheless, given the magnitude of sedimentary OC stocks relative to corresponding soil carbon stocks revealed by the present investigation, the question arises as to whether land-proximal ocean sediments underlying territorial waters should be considered as part of national carbon accounting and potential GHG mitigation projects and subject to management against human-induced disturbance. Although the nature of anthropogenic interactions with coastal ocean ecosystems is fundamentally different from that on land, there is undeniable impact of humans over the entire globe, and myriad ways that humans directly and indirectly impact the ocean carbon cycle. In the most recent IPCC report [28], the carbon stock inventory framework focuses on wet and drained soils as well as managed wetlands, which share similarities of processes at the land-sea interface [9]. This issue may be especially pertinent for nations and associated territories with large EEZ-to-land area ratios, where existing sediment carbon stocks could have a large impact on inventories. For countries with upwelling zones and relative low soil carbon stocks, such as Namibia, marine sediment stocks can be equally relevant. Carbon stocks of nations such as Chile, where its EEZ accounts for a major fraction (73%; Additional file 1: Table S1) of its overall territory and that hosts extensive fjords which are recognized as globally significant hotspots of OC burial [52], may indeed be dominated by carbon residing in the marine realm. Even for countries with relatively low EEZ-land area ratios such as Pakistan (26%), we find that sediment stocks can constitute a significant fraction of total carbon stocks.

Coastal areas offer a wealth of economically valuable resources, provide diverse ecosystem services, and have in general been locations of rapid development and intensive land use. Coastal systems are also very sensitive to change [7], particularly at the land–ocean interface, and receive enormous quantities of terrigenous materials, support important and yet fragile ecosystems such as mangroves, marshes and coral reefs, and both sequester and export large amounts of carbon [59].

In addition to the magnitude of carbon stocks, the rate of change (accumulation, attrition) of these stocks is of key concern with respect to their ability to ameliorate or exacerbate changes in atmospheric carbon dioxide levels, which in turn relates to sediment accumulation/soil formation rates. The rate of carbon build-up in soils versus those in continental margin sediments are of a similar order: it takes up to 1000 years for around 2.5 cm (1 inch) of topsoil to develop [58], while sediment accumulation rates on continental shelves and upper slopes typically range from a few cm to several tens of cm per 1000 years [37]. Organic carbon is generally highest in these upper soil and sediment layers, and support rich and diverse biological communities. Losses of soil (carbon) through enhanced erosion and other processes are the subject of considerable concern, and discussed in the context of diminishing soil fertility [44]. In contrast, potential changes to sediment carbon stocks are much less well studied or understood, but may also be significant in the face of exploitation of marine resources as well as changing fluxes from land and changing ocean conditions (deoxygenation, warming, acidification), may in turn influence marine productivity and carbon burial [30].

Given that an increasing number of countries are implementing policies to mitigate climate change, this study raises the question of whether a more comprehensive accounting of carbon stocks that includes human activities in both terrestrial and oceanic realms is necessary to ensure environmental and ecological integrity in both terrestrial and marine domains of the earth system. A major challenge lies in the integration of political and economic principles with those of ecosystem conservation for effective stewardship of resources of coastal margins, especially within EEZs [33]. Overall, when we evaluate the implications of extending existing carbon emission trade markets to include the carbon sequestration in the ocean for maritime nations, the future economic ramifications may be considerable, especially for nations with high EEZ-land ratios and of those that host rich deposits of marine sedimentary carbon. From a global perspective, the construction of databases of oceanic sediment carbon concentrations and associated physical properties would be essential for better understanding and assessing the potential oceanic carbon sequestration, as well as potential threats of carbon loss.

## Conclusions

We compared approximated organic carbon stocks of adjacent continental margin sediments within corresponding Exclusive Economic Zones (EEZs) with those in soils for three maritime countries. This initial comparison reveals that sediments of some maritime nations may hold carbon stocks that approach or exceed those of corresponding soils. The magnitude of estimated ocean carbon stocks brings into focus questions concerning their importance to and impact on current GHG reporting activities.

In addition to gauging carbon stocks in continental versus oceanic reservoirs, assessing the transfer of carbon between terrestrial and marine realms is crucial for advancing knowledge of biogeochemical cycles and on ecosystem functioning, with both issues of potential relevance to design of future carbon accounting of maritime nations. Taking into account carbon sequestration and release in both terrestrial as well as the aquatic environments is pivotal for development of comprehensive climate mitigation strategies [6].

From both a practical and political perspective, the premise of using EEZ of maritime nations as a means to define marine sediment carbon stocks may be a fair and legitimate approach. Quantifying the potential for carbon sequestration, or carbon release, from coastal regions may provide motivation for implementation of policies in developed and especially in developing countries that combat ecosystem degradation, whilst promoting a more sustainable use and conservation of their natural resources. Thus, appropriate carbon stock assessment of maritime nations might positively affect coastal management strategies and promote protection of coastal ecosystems. In view of the projected growth of the global carbon trade market, the quantification of carbon sequestration of maritime nations may thus grow in economic importance, as in the future there might be ways of getting carbon credits when marine activities were stopped.

Significant conceptual and practical challenges are associated with development of carbon asset accounts. This study does not attempt to provide a solution to such challenges, or to provide advice or recommendations concerning specific implementation strategies. Instead, our contribution is to show the relevance of marine sediment carbon measures, which could have an impact on the management and accounting of human activities in the context of GHG inventories. Our exploratory study highlights the importance of the availability of extensive and reliable data in order to enhance the scientific and technical basis for further discussions on the carbon stocks in both oceanic and terrestrial realms. Our further work includes collecting geochemical data of marine sediments from the scientific literature to establish a database, analysis and use of appropriate interpolation methods to gain information for ocean regions with sparse sediment data available, as well as the estimation and comparison of carbon stocks of more maritime nations. Such comprehensive data measurements and collection of relevant data are of great value for improved understanding of the global carbon cycle, for further assessment of human activities impacting carbon stocks of coastal areas, and ultimately for the development of informed policies supporting decisions concerning the future stewardship of our planet.

## Additional file

**Additional file 1.** Supplementary material with additional figure S1 and table S1.
