# Supplementary material for: Relevance of carbon stocks of marine sediments for national greenhouse gas inventories of maritime nations
Source: Carbon Balance Manag. 2017 May 10;12:10. doi: 10.1186/s13021-017-0077-x (PMC5423874; doi:10.1186/s13021-017-0077-x)
Supplement: Supplementary file 1 — Additional file 1. Supplementary material with additional figure S1 and table S1. [file 13021_2017_77_MOESM1_ESM.doc]

**Supplementary Material:**

**Exclusive Economical Zone (EEZ)**

The 1982 United Nations Convention on Law of the Sea (UNCLOS) gave coastal states permanent sovereignty over natural resources found in the waters superjacent to their seabed and in their subsoil (i.e., sediment). This new order on the seas presented countries with an opportunity to conserve their fish stocks and assess possibilities for economic exploration and exploitation of other natural resources, such as oil, gas and minerals, off their coasts. The outer limit of the Exclusive Economic Zone shall not exceed 200 nautical miles (370.4 km) from the baselines against from which the breadth of the territorial sea is measured (UNCLOS, 2015). Countries are allowed to claim a further extension of the continental shelf up to 350 nautical miles (648.2 km) from the baselines of the territorial sea, if they can prove that the claimed area constituted a natural prolongation of their land territory. Figure S1 presents all nations and the corresponding EEZ limits (see also Supplemental Table S1).


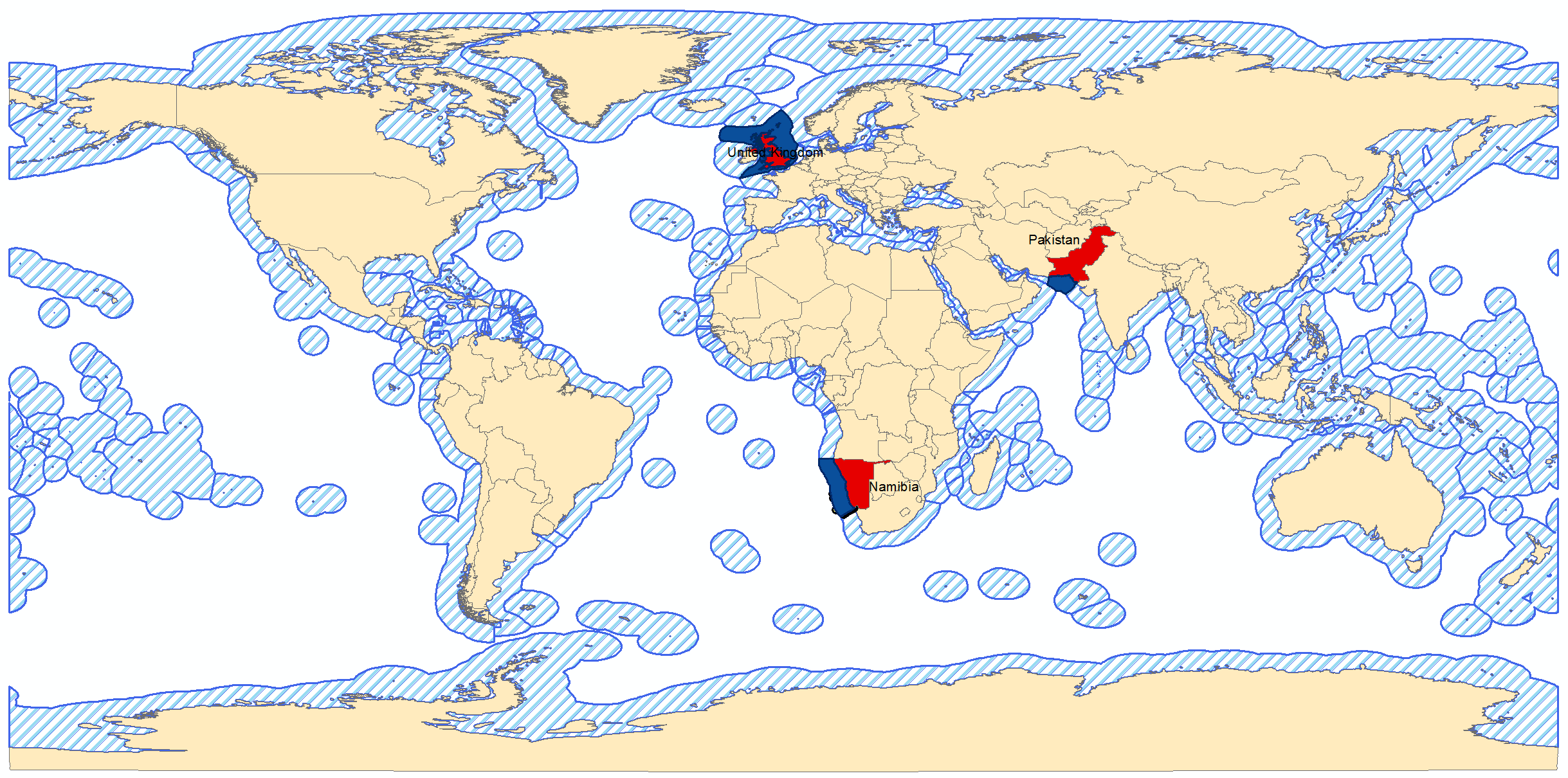


**Fig. S1.** World Exclusive Economic Zones (EEZs; hatched blue). National boundaries of Namibia, Pakistan, United Kingdom (red) and their respective EEZs (dark blue) are highlighted.

**International climate agreements**

The Kyoto Protocol is linked to the United Nations Framework Convention on Climate Change (UNFCCC) and aims to combat climate change by reducing greenhouse gas emissions. The agreement was adopted in 1997, came into effect in 2005 and will be in effect until 2020 (UNFCCC, 2016). 192 countries ratified it, including the three countries considered in this study. An integral part of this agreement is that the emission, and conversely sequestration, of greenhouse gases (GHG) gains an economic value. More specifically, countries with mitigation commitments under the Kyoto Protocol use crediting mechanisms to generate and transfer Emission Reduction Units (ERUs) from Greenhouse Gas abatement projects. From 2020 onwards, the new Paris Agreement will come into effect for 195 countries that have so far ratified it. The Paris agreement involves comparatively more ambitious targets that are expected to further strengthen and expand the carbon market (EC, 2015).

**Namibia**

##### The EEZ area of Namibia extends 200 nautical miles distant from its continental territory and makes up a total of 560,101 km^2^ (SAU, 2016). This maritime area constitutes an economically valuable fishery particularly within the Benguela upwelling system along the coast (UNEP, 2006). Other major marine resources of potential expansion are the off-shore Kudu gas fields, off-shore diamond mining and phosphorite (fertilizer) deposits (Compton and Bergh, 2016).

The areal extent of Namibian land is 823,290 km^2^ (TWB, 2016). With respect to continental organic carbon (OC) stocks, low rainfall and poor soils characterize most of this semi-arid to arid country that is dominated by deserts, savannas and dry woodlands (FAO, 2016). In contrast to the relatively barren landmass, the adjacent Namibian continental margin is characterized by very high biological productivity fueled by coastal upwelling of nutrient-rich waters. This results in a pronounced and extensive oxygen deficient zone and elevated OC contents in underlying sediments.

The average carbon content in the topsoil (0-10 cm) was obtained from the statistics of the Food and Agriculture Organization (FAO, 2008), which is based on national gridded values provided by the Harmonized World Soil Database (HWSD) (Nachtergaele et al., 2012). Sediment OC data were extracted from the GSA Data Repository item 2006038 (Inthorn et al., 2006), in which 968 samples were retrieved at 95 stations extending from the sediment-water interface to approximately 30 cm below the seafloor (see Fig. 4a). Mean OC values for topsoils and marine sediments are 0.34% (FAO, 2008) and 3.71% (Inthorn et al., 2006), respectively. Corresponding mean BD values are 1.55 kg/dm3 for soil (0-30 cm) (Leenaars et al., 2014) and 1.17 g/cm3 (Filipelli, 1997) for sediment (0-10 cm).

**Pakistan**

Important oceanic phenomena and features characterize the EEZ of Pakistan, such as monsoonal climate, upwelling and terrigenous inputs from the Indus River (NIOPK, 2016). Besides supporting fisheries, Pakistan’s seabed territory may also host sub-sea oil and gas deposits. In 2015, the United Nations’ Commission on Limits of Continental Shelf (UNCLCS) accepted Pakistan’s claim for extending the EEZ limits by 150 nautical miles. Thus, Pakistan’s EEZ of about 222,255 km^2^ (SAU, 2016) has an additional shelf area of about 50,000 km^2^.

The areal extent of Pakistan land is 770,880 km^2^ (TWB, 2016). Pakistan is prone to extremes in weather and climate, with frequent floods and droughts, and associated severe challenges with respect to supply of safe drinking water and sanitation. The soils in Pakistan’s dry interior are generally low in organic matter - the reported range of soil organic carbon (SOC) is from 0.52% to 1.38% (Azam et al., 2001).

In this study, the mean organic carbon in the topsoil of Pakistan was obtained from FAO (2008). The Pakistan margin sediment data consisted of 31 samples found in the PANGAEA data repository (Schulte et al., 2000; Fig. 4b), 5 samples from Cowie et al. (2009), 26 samples from Cowie et al. (1999) and 47 samples from Suthof et al. (2000). For the soils of Pakistan, BD values vary from 1.3 to 2.6 g/cm^3^, according to the World Bulk Density Map (IGBP, 2015). Mean OC values for top soils (0-10 cm) and sediments (0-10 cm) for Pakistan and its EEZ are 0.86% (FAO, 2008) and 1.82% (Schulte et al., 2000), respectively; corresponding mean soil and sediment BD values are 1.5 g/cm^3^ (IGBP, 2015) and 0.8 g/cm^3^ (Cowie et al., 1999).

**United Kingdom**

The United Kingdom of Great Britain and Northern Ireland (UK) is surrounded by a relatively large adjacent continental shelf, which has been widely surveyed (e.g., Graham and Trotmam, 1986). The UK’s EEZ area, without its dependencies and oversea territories, is about 756,639 km^2^ (SAU, 2016).

The land area of UK is 241,930 km^2^ (TWB, 2016). In contrast to the monsoonal climate of Pakistan and the generally dry climate of Namibia, rainfall in the UK is relatively high and uniformly distributed throughout the year. A temperate climate and relatively high annual levels of precipitation support high organic carbon contents in UK soils. Until 2003, no reliable inventory of soil organic carbon for the UK existed, as required by the Kyoto Protocol. Nowadays, various databases are available for UK topsoils and subsoils (e.g., LUCAS, Tóth et al., 2013; Bradley et al., 2005).

For consistency in this study, the mean organic carbon of topsoil in the UK was obtained from FAO statistics (FAO, 2008). Corresponding marine sediment data (102 samples) were found in Loh et al. (2008), Gough et al. (1993), Gontikaki (2013), de Haas et al. (1997) and Serpetti et al. (2012) (see Fig. 4c). BD values were obtained from the British Oceanographic Data Centre (BDOC). Mean OC values for topsoils and sediments in the UK are 6.98% (FAO, 2008) and 2.32% (Loh et al., 2008; Gough et al., 1993; Gontikaki et al., 2013), respectively. Corresponding mean BD are 0.73 g/cm^3^ (UKSO, 2015) and 1.13 g/cm^3^ (BDOC, 2015), respectively.

**Table S1.** Maritime nations and various islands with EEZ area, land area and EEZ proportion of total area (EEZ plus land areas). Data were collected from several open sources, mainly SAU (2016) and TWB (2016).

| **Maritime Nation** | **Sovereign** | **EEZ (km^2^)** | **Land area (km^2^)** | **EEZ proportion of total area (%)** | |
| --- | --- | --- | --- | --- | --- |
| Albania | Albania | 11'105 | 27'400 | 29 |  |
| Algeria | Algeria | 128'843 | 2'381'740 | 5 |  |
| American Samoa | United States | 404'367 | 200 | 100 |  |
| Amsterdam Island and Saint Paul Island | France | 508'763 | 6'290 | 99 |  |
| Andaman and Nicobar | India | 659'590 | 8'249 | 99 |  |
| Angola | Angola | 490'684 | 1'246'700 | 28 |  |
| Anguilla | United Kingdom | 92'178 | 74 | 100 |  |
| Antarctica | Antarctica | 10'233'940 | 12'277'658 | 45 |  |
| Antigua and Barbuda | Antigua and Barbuda | 107'939 | 440 | 100 |  |
| Argentina | Argentina | 1'082'467 | 2'736'690 | 28 |  |
| Aruba | Netherlands | 25'199 | 180 | 99 |  |
| Ascension | United Kingdom | 441'642 | 91 | 100 |  |
| Australia | Australia | 6'369'268 | 7'682'300 | 45 |  |
| Azerbaijan | Azerbaijan | 77'059 | 82'659 | 48 |  |
| Azores | Portugal | 955'644 | 2'346 | 100 |  |
| Bahamas | Bahamas | 628'026 | 10'010 | 98 |  |
| Bahrain | Bahrain | 8'826 | 770 | 92 |  |
| Balearic Island | Balearic Island | 128'053 | 4'992 | 96 |  |
| Bangladesh | Bangladesh | 84'846 | 130'170 | 39 |  |
| Barbados | Barbados | 183'773 | 430 | 100 |  |
| Bassas da India | France | 121'906 | 6'290 | 95 |  |
| Belgium | Belgium | 3'479 | 30'280 | 10 |  |
| Belize | Belize | 36'182 | 22'810 | 61 |  |
| Benin | Benin | 30'286 | 112'760 | 21 |  |
| Bermuda | United Kingdom | 450'347 | 50 | 100 |  |
| Bonaire | Netherlands | 13'218 | 294 | 98 |  |
| Bosnia and Herzegovina | Bosnia and Herzegovina | 14 | 51'200 | 0 |  |
| Bouvet Island | Norway | 441'174 | 49 | 100 |  |
| Brazil | Brazil | 2'400'918 | 8'358'140 | 22 |  |
| British Indian Ocean Territory | United Kingdom | 641'284 | 170 | 100 |  |
| British Virgin Islands | United Kingdom | 80'111 | 150 | 100 |  |
| Brunei | Brunei | 25'340 | 5'270 | 83 |  |
| Bulgaria | Bulgaria | 35'132 | 108'560 | 24 |  |
| Cambodia | Cambodia | 47'676 | 176'520 | 21 |  |
| Cameroon | Cameroon | 14'669 | 472'710 | 3 |  |
| Canada | Canada | 5'768'187 | 9'093'510 | 39 |  |
| Canary Islands | Spain | 455'328 | 503'250 | 48 |  |
| Cape Verde | Cape Verde | 796'555 | 4'033 | 99 |  |
| Cayman Islands | United Kingdom | 119'134 | 240 | 100 |  |
| Chile | Chile | 1'975'168 | 743'532 | 73 |  |
| China | China | 2'236'430 | 9'388'211 | 19 |  |
| Christmas Island | Australia | 327'992 | 99 | 100 |  |
| Clipperton Island | France | 431'273 | 9 | 100 |  |
| Cocos Islands | Australia | 467'229 | 10 | 100 |  |
| Colombia | Colombia | 744'317 | 1'109'500 | 40 |  |
| Colombia | Colombia | 180'000 | 56 | 100 |  |
| Comoro Islands | Comoro Islands | 165'181 | 1'119 | 99 |  |
| Congo | Congo | 39'618 | 341'500 | 10 |  |
| Congo, DRC | Congo, DRC | 13'140 | 2'267'050 | 1 |  |
| Cook Islands | New Zealand | 1'960'027 | 55 | 100 |  |
| Cote d'Ivoire | Cote d'Ivoire | 173'764 | 318'000 | 35 |  |
| Costa Rica | Costa Rica | 572'131 | 51'060 | 92 |  |
| Crete | Greece | 96'568 | 8'336 | 92 |  |
| Croatia | Croatia | 55'961 | 55'960 | 50 |  |
| Crozet Islands | France | 574'539 | 6'290 | 99 |  |
| Cuba | Cuba | 364'511 | 106'449 | 77 |  |
| Curacao | Netherlands | 30'427 | 444 | 99 |  |
| Cyprus | Cyprus | 98'088 | 9'240 | 91 |  |
| Dem. Rep. of Congo | Dem. Rep. of Congo | 13'140 | 2'267'050 | 1 |  |
| Denmark | Denmark | 102'693 | 42'430 | 71 |  |
| Djibouti | Djibouti | 7'037 | 23'180 | 23 |  |
| Dominica | Dominica | 28'593 | 750 | 97 |  |
| Dominican Republic | Dominican Republic | 269'489 | 48'320 | 85 |  |
| East Timor | East Timor | 77'051 | 15'496 | 83 |  |
| Easter Island | Chile | 720'410 | 722'511 | 50 |  |
| Ecuador | Ecuador | 236'556 | 248'360 | 49 |  |
| Egypt | Egypt | 260'404 | 995'450 | 21 |  |
| El Salvador | El Salvador | 93'713 | 20'850 | 82 |  |
| Equatorial Guinea | Equatorial Guinea | 308'275 | 28'050 | 92 |  |
| Eritrea | Eritrea | 78'383 | 101'000 | 44 |  |
| Estonia | Estonia | 36'512 | 42'390 | 46 |  |
| Faeroe Islands | Denmark | 267'833 | 1'396 | 99 |  |
| Falkland Islands | United Kingdom | 549'974 | 10'217 | 98 |  |
| Fernando de Noronha | Brazil | 363'373 | 26 | 100 |  |
| Fiji | Fiji | 1'281'703 | 18'270 | 99 |  |
| Finland | Finland | 81'522 | 303'890 | 21 |  |
| France | France | 309'858 | 547'557 | 36 |  |
| French Guiana | France | 134'656 | 83'726 | 62 |  |
| French Polynesia | France | 4'771'088 | 4'167 | 100 |  |
| Gabon | Gabon | 191'944 | 257'670 | 43 |  |
| Galapagos Islands | Ecuador | 835'541 | 254'767 | 77 |  |
| Gambia | Gambia | 22'650 | 10'120 | 69 |  |
| Georgia | Georgia | 22'947 | 69'490 | 25 |  |
| Germany | Germany | 56'464 | 348'540 | 14 |  |
| Ghana | Ghana | 225'661 | 227'540 | 50 |  |
| Gibraltar | United Kingdom | 426 | 10 | 98 |  |
| Glorieuse Islands | France | 43'430 | 6'290 | 87 |  |
| Greece | Greece | 397'140 | 128'900 | 75 |  |
| Greenland | Denmark | 2'278'113 | 410'450 | 85 |  |
| Grenada | Grenada | 26'133 | 340 | 99 |  |
| Guadeloupe | France | 90'570 | 1'120 | 99 |  |
| Guatemala | Guatemala | 117'676 | 107'160 | 52 |  |
| Guernsey | United Kingdom | 8'666 | 78 | 99 |  |
| Guinea | Guinea | 109'439 | 245'720 | 31 |  |
| Guinea Bissau | Guinea Bissau | 105'839 | 28'120 | 79 |  |
| Guyana | Guyana | 140'369 | 196'850 | 42 |  |
| Haiti | Haiti | 123'525 | 27'560 | 82 |  |
| Hawaii | United States | 2'474'682 | 28'311 | 99 |  |
| Heard and McDonald Islands | Australia | 416'973 | 368 | 100 |  |
| Honduras | Honduras | 218'804 | 111'890 | 66 |  |
| Howland and Baker Island | United States | 434'922 | 5 | 100 |  |
| Iceland | Iceland | 756'112 | 100'250 | 88 |  |
| Ile Europa | France | 125'358 | 28 | 100 |  |
| Ile Tromelin | France | 270'477 | 1 | 100 |  |
| India | India | 1'629'607 | 2'973'190 | 35 |  |
| Indonesia | Indonesia | 6'024'450 | 1'811'570 | 77 |  |
| Iran | Iran | 163'730 | 1'628'550 | 9 |  |
| Iraq | Iraq | 540 | 434'320 | 0 |  |
| Ireland | Ireland | 409'929 | 68'890 | 86 |  |
| Israel | Israel | 25'170 | 21'640 | 54 |  |
| Italy | Italy | 315'943 | 294'140 | 52 |  |
| Jamaica | Jamaica | 263'284 | 10'830 | 96 |  |
| Jan Mayen | Norway | 292'189 | 373 | 100 |  |
| Japan | Japan | 4'464'772 | 364'560 | 92 |  |
| Jarvis Island | United States | 316'555 | 5 | 100 |  |
| Jersey | United Kingdom | 2'956 | 120 | 96 |  |
| Johnston Atoll | United States | 442'629 | 3 | 100 |  |
| Jordan | Jordan | 97 | 88'780 | 0 |  |
| Juan de Nova Island | France | 62'416 | 6'290 | 91 |  |
| Kazakhstan | Kazakhstan | 119'013 | 2'699'700 | 4 |  |
| Kenya | Kenya | 162'794 | 569'140 | 22 |  |
| Kerguelen Islands | France | 567'687 | 6'290 | 99 |  |
| Kiribati | Kiribati | 1'048'697 | 810 | 100 |  |
| Kuwait | Kuwait | 11'786 | 17'820 | 40 |  |
| Latvia | Latvia | 28'986 | 62'190 | 32 |  |
| Lebanon | Lebanon | 19'265 | 10'230 | 65 |  |
| Liberia | Liberia | 246'093 | 95'659 | 72 |  |
| Libya | Libya | 355'604 | 1'759'540 | 17 |  |
| Line Islands | Kiribati | 1'645'373 | 267 | 100 |  |
| Lithuania | Lithuania | 6'139 | 62'675 | 9 |  |
| Macquarie Island | Australia | 477'325 | 7'694'273 | 6 |  |
| Madagascar | Madagascar | 1'200'330 | 581'800 | 67 |  |
| Madeira | Portugal | 454'459 | 801 | 100 |  |
| Malaysia | Malaysia | 449'477 | 328'550 | 58 |  |
| Maldives | Maldives | 916'011 | 300 | 100 |  |
| Malta | Malta | 55'542 | 320 | 99 |  |
| Marshall Islands | Marshall Islands | 1'992'022 | 180 | 100 |  |
| Martinique | France | 47'372 | 780 | 98 |  |
| Mauritania | Mauritania | 204'596 | 1'030'700 | 17 |  |
| Mauritius | Mauritius | 1'276'958 | 2'030 | 100 |  |
| Mayotte | France | 62'982 | 374 | 99 |  |
| Mexico | Mexico | 3'273'549 | 1'943'950 | 63 |  |
| Micronesia | Micronesia | 2'992'415 | 700 | 100 |  |
| Monaco | Monaco | 286 | 2 | 99 |  |
| Montenegro | Montenegro | 7'460 | 13'450 | 36 |  |
| Montserrat | United Kingdom | 7'586 | 77 | 99 |  |
| Morocco | Morocco | 577'068 | 446'300 | 56 |  |
| Mozambique | Mozambique | 571'452 | 786'380 | 42 |  |
| Myanmar | Myanmar | 511'389 | 653'080 | 44 |  |
| Namibia | Namibia | 560'101 | 823'290 | 40 |  |
| Nauru | Nauru | 308'506 | 21 | 100 |  |
| Netherlands | Netherlands | 61'869 | 33'670 | 65 |  |
| New Caledonia | France | 1'422'596 | 18'576 | 99 |  |
| New Zealand | New Zealand | 3'479'752 | 263'310 | 93 |  |
| Nicaragua | Nicaragua | 222'754 | 120'340 | 65 |  |
| Nigeria | Nigeria | 216'325 | 910'770 | 19 |  |
| Niue | New Zealand | 316'584 | 180 | 100 |  |
| Norfolk Island | Australia | 430'824 | 39 | 100 |  |
| North Korea | North Korea | 115'667 | 120'410 | 49 |  |
| Northern Mariana Islands and Guam | United States | 748'867 | 460 | 100 |  |
| Northern Saint-Martin | France | 5'456 | 54 | 99 |  |
| Norway | Norway | 935'397 | 365'245 | 72 |  |
| Oecusse Ambeno | East Timor | 2'270 | 15'496 | 13 |  |
| Oman | Oman | 529'559 | 309'500 | 63 |  |
| Pakistan | Pakistan | 272'255 | 770'880 | 26 |  |
| Palau | Palau | 604'253 | 460 | 100 |  |
| Palmyra Atoll | United States | 352'526 | 12 | 100 |  |
| Panama | Panama | 330'783 | 74'340 | 82 |  |
| Papua New Guinea | Papua New Guinea | 2'396'575 | 452'860 | 84 |  |
| Paracel Islands | Disputed | 294'013 | 8 | 100 |  |
| Peru | Peru | 856'955 | 1'280'000 | 40 |  |
| Philippines | Philippines | 2'263'816 | 298'170 | 88 |  |
| Phoenix Islands | Kiribati | 743'062 | 267 | 100 |  |
| Pitcairn | United Kingdom | 836'103 | 28 | 100 |  |
| Poland | Poland | 32'058 | 306'210 | 9 |  |
| Portugal | Portugal | 311'648 | 91'600 | 77 |  |
| Prince Edward Islands | South Africa | 473'371 | 1'219'930 | 28 |  |
| Puerto Rico | United States | 177'327 | 8'870 | 95 |  |
| Qatar | Qatar | 31'819 | 11'610 | 73 |  |
| Republique du Congo | Republique du Congo | 39'754 | 341'500 | 10 |  |
| Reunion | France | 315'071 | 2'512 | 99 |  |
| Romania | Romania | 29'756 | 230'030 | 11 |  |
| Russia | Russia | 7'636'834 | 16'376'870 | 32 |  |
| Saba | Netherlands | 9'470 | 13 | 100 |  |
| Saint Helena | United Kingdom | 444'898 | 123 | 100 |  |
| Saint Kitts and Nevis | Saint Kitts and Nevis | 10'209 | 260 | 98 |  |
| Saint Lucia | Saint Lucia | 15'472 | 616 | 96 |  |
| Saint Pierre and Miquelon | France | 12'353 | 242 | 98 |  |
| Saint Vincent and the Grenadines | Saint Vincent and the Grenadines | 36'304 | 389 | 99 |  |
| Samoa | Samoa | 131'535 | 2'830 | 98 |  |
| Sao Tome and Principe | Sao Tome and Principe | 165'345 | 960 | 99 |  |
| Sardinia | Italy | 117'400 | 24'090 | 83 |  |
| Saudi Arabia | Saudi Arabia | 220'184 | 2'149'690 | 9 |  |
| Senegal | Senegal | 157'709 | 192'530 | 45 |  |
| Seychelles | Seychelles | 1'331'964 | 455 | 100 |  |
| Sierra Leone | Sierra Leone | 159'300 | 72'180 | 69 |  |
| Singapore | Singapore | 673 | 707 | 49 |  |
| Sint-Eustasius | Netherlands | 2'260 | 21 | 99 |  |
| Sint-Maarten | Netherlands | 499 | 87 | 85 |  |
| Slovenia | Slovenia | 192 | 20'140 | 1 |  |
| Solomon Islands | Solomon Islands | 1'596'464 | 27'990 | 98 |  |
| Somalia | Somalia | 831'059 | 627'340 | 57 |  |
| South Africa | South Africa | 1'065'941 | 1'213'090 | 47 |  |
| South Georgia and the South Sandwich Islands | United Kingdom | 1'203'155 | 3'416 | 100 |  |
| South Korea | South Korea | 473'280 | 96'460 | 83 |  |
| Southern Kuriles | Disputed | 214'148 | 2'223 | 99 |  |
| Spain | Spain | 437'458 | 500'210 | 47 |  |
| Spratly Islands | Disputed | 440'758 | 4 | 100 |  |
| Sri Lanka | Sri Lanka | 530'945 | 62'710 | 89 |  |
| Sudan | Sudan | 92'513 | 2'376'000 | 4 |  |
| Suriname | Suriname | 127'817 | 156'000 | 45 |  |
| Svalbard | Norway | 804'907 | 470 | 100 |  |
| Sweden | Sweden | 156'296 | 407'340 | 28 |  |
| Syria | Syria | 10'189 | 183'630 | 5 |  |
| Taiwan | Taiwan | 1'148'485 | 36'193 | 97 |  |
| Tanzania | Tanzania | 241'129 | 885'800 | 21 |  |
| Thailand | Thailand | 305'778 | 510'890 | 37 |  |
| Togo | Togo | 15'442 | 54'390 | 22 |  |
| Tokelau | New Zealand | 319'049 | 10 | 100 |  |
| Tonga | Tonga | 664'751 | 720 | 100 |  |
| Trindade and Mart.Vaz | Brazil | 468'599 | 10 | 100 |  |
| Trinidad and Tobago | Trinidad and Tobago | 79'798 | 5'131 | 94 |  |
| Tristan da Cunha | United Kingdom | 754'306 | 207 | 100 |  |
| Tunisia | Tunisia | 102'047 | 155'360 | 40 |  |
| Turkey | Turkey | 256'332 | 769'630 | 25 |  |
| Turkmenistan | Turkmenistan | 79'582 | 469'930 | 14 |  |
| Turks and Caicos Islands | United Kingdom | 153'533 | 950 | 99 |  |
| Tuvalu | Tuvalu | 751'672 | 30 | 100 |  |
| Ukraine | Ukraine | 132'414 | 579'320 | 19 |  |
| United Arab Emirates | United Arab Emirates | 56'826 | 83'600 | 40 |  |
| United Kingdom | United Kingdom | 756'639 | 241'930 | 76 |  |
| United States | United States | 2'451'460 | 9'147'420 | 21 |  |
| Uruguay | Uruguay | 133'014 | 175'020 | 43 |  |
| Vanuatu | Vanuatu | 827'626 | 12'190 | 99 |  |
| Venezuela | Venezuela | 474'769 | 882'050 | 35 |  |
| Vietnam | Vietnam | 1'395'096 | 310'070 | 82 |  |
| Virgin Islands of the U.S. | United States | 33'748 | 350 | 99 |  |
| Wake Island | United States | 407'238 | 7 | 100 |  |
| Wallis and Futuna | France | 258'270 | 55 | 100 |  |
| Western Sahara | Morocco | 251'564 | 268'179 | 48 |  |
| Yemen | Yemen | 545'101 | 527'970 | 51 |  |

**Additional references** (not cited in the main manuscript):

Azam, F., Iqbal, M.M., Inayatullah, C., Malik, K.A. Technologies for Sustainable Agriculture. Nuclear Institute for Agriculture and Biology, Faisalabad. 2001. 144 pages.

Bradley, R.I., Milne, R., Bell, J., Lilly, A., Jordan, C., Higgins, A. A soil carbon and land use database for the United Kingdom, Soil Use and Management 2005; 21:363-369.

Compton J.S. and Bergh, E.W. Phosphorite deposits on the Namibian shelf. Marine Geology 2016; 380:290-314.

FAO (2016) Food and Agriculture Organization of the United Nations. *http://www. fao.org/ag/agp/agpc/doc/counprof/namibia.htm*. Accessed on Feb 2016.

Graham and Trotman (Eds) Exclusive Economic Zones - Resources, Opportunities and the Legal Regime. Proc. of international conference, Advances in underwater technology and offshore engineering 1986; vol. 8, London, UK.

NIOPK. National Institute of Oceanography, Ministry of Science & Technology, Government of Pakistan. *http://www.niopk.gov.pk/intro-1.html*. Accessed on Mar 2016.

SAU. Sea Around Us. 2016. *http://www.seaaroundus.org/data/#/eez*. Accessed on July. 2016.

TWB. The World Bank. 2016. *http://data.worldbank.org/indicator/AG.LND.TOTL.K2*. Accessed on July.2016.

UNCLOS. United National Convention on the Law of the Sea. 2015. *http://www.un.org/ depts/los/convention_agreements/texts/unclos/part5.htm*. Accessed on September.2015

UNEP. United Nations Environment Programme. Africa Environment Outlook 2 – Our Environment, Our Wealth. 2006. *http://www.unep.org/DEWA/Africa/AEO2_Launch/*. Accessed on July.2015.

UNFCCC. United Nations Framework Convention on Climate Change, The Kyoto Protocol. Technical Report 2007. *http://unfccc.int/resource/docs/publications/ mechanisms.pdf*. Accessed on Sep 2015.

UNFCCC. United Nations Framework Convention on Climate Change, System of Environmental Economic Accounting Central Framework. Technical Report 2012. http://unstats.un.org/unsd/envaccounting/seeaRev/SEEA_CF_Final_en.pdf. Accessed on Sep 2015.

UNFCCC. United Nations Framework Convention on Climate Change, System of Environmental Economic Accounting Central Framework. 2016. http://unfccc.int/kyoto_ protocol/status_of_ratification/items/2613.php. Accessed on Jan 2015.
